# Spectroscopically Orthogonal Spin Labels in Structural Biology at Physiological Temperatures

**DOI:** 10.1021/acs.jpcb.3c04497

**Published:** 2023-07-25

**Authors:** Markus Teucher, Svetlana Kucher, M. Hadi Timachi, C. Blake Wilson, Dariusz Śmiłowicz, Raphael Stoll, Nils Metzler-Nolte, Mark S. Sherwin, Songi Han, Enrica Bordignon

**Affiliations:** † Faculty of Chemistry and Biochemistry, Ruhr University of Bochum, Bochum 44801, Germany; ‡ Department of Physical Chemistry, University of Geneva, Genève 1211, Switzerland; § Department of Physics, University of California, Santa Barbara, Santa Barbara, California 93106, United States; ∥ Institute for Terahertz Science and Technology, University of California, Santa Barbara, Santa Barbara, California 93106, United States; ⊥ Department of Chemistry and Biochemistry, University of California, Santa Barbara**,** Santa Barbara, California 93106, United States

## Abstract

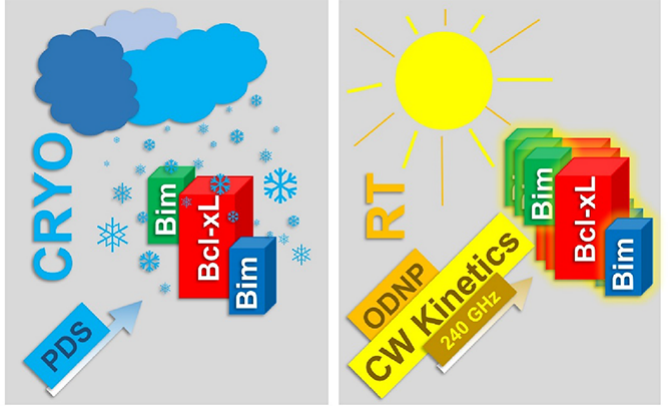

Electron paramagnetic resonance spectroscopy (EPR) is mostly used in structural biology in conjunction with pulsed dipolar spectroscopy (PDS) methods to monitor interspin distances in biomacromolecules at cryogenic temperatures both in vitro and in cells. In this context, spectroscopically orthogonal spin labels were shown to increase the information content that can be gained per sample. Here, we exploit the characteristic properties of gadolinium and nitroxide spin labels at physiological temperatures to study side chain dynamics via continuous wave (cw) EPR at X band, surface water dynamics via Overhauser dynamic nuclear polarization at X band and short-range distances via cw EPR at high fields. The presented approaches further increase the accessible information content on biomolecules tagged with orthogonal labels providing insights into molecular interactions and dynamic equilibria that are only revealed under physiological conditions.

## Introduction

**Figure 1:**
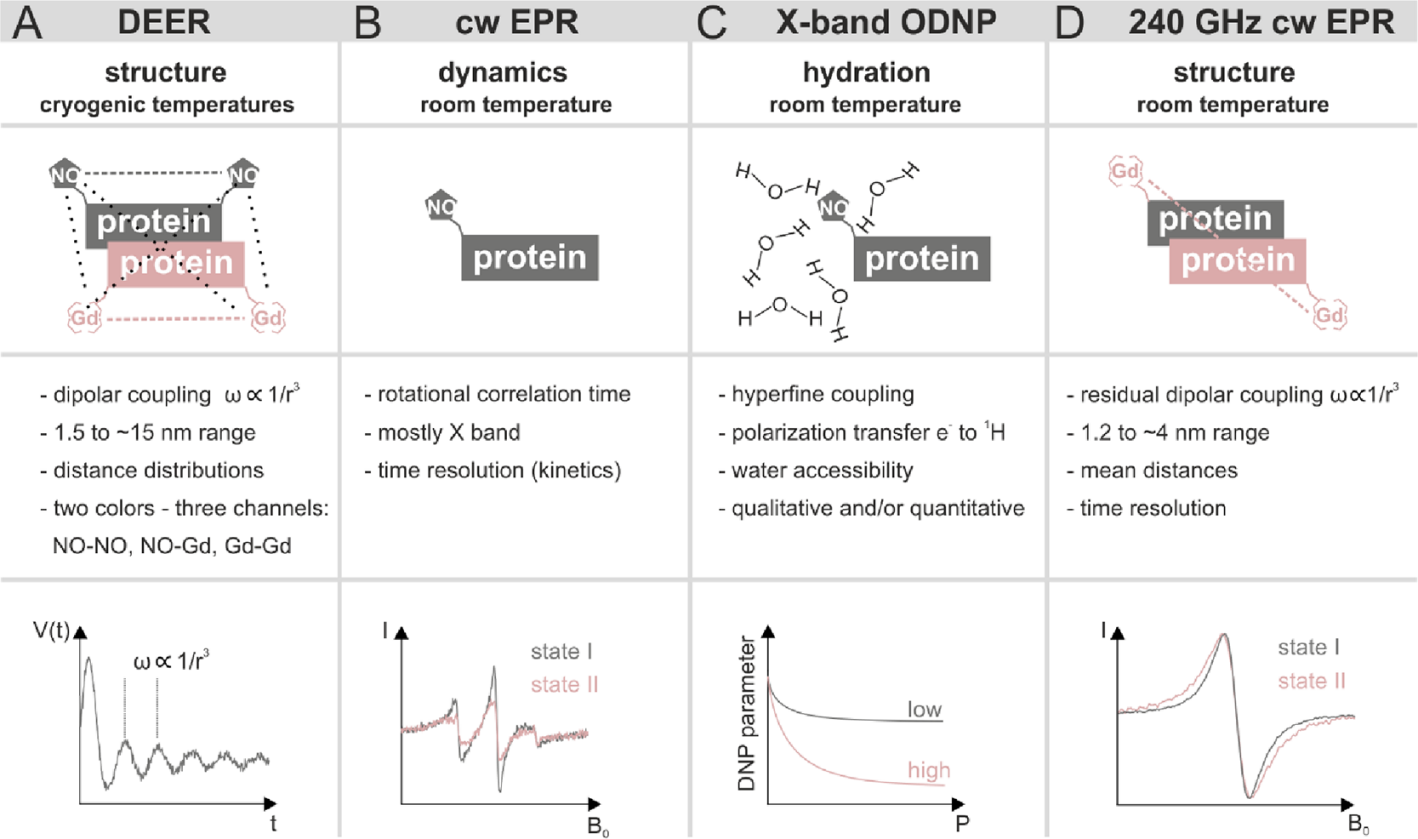
DEER vs room temperature methods. Summary of the most relevant information, which can be extracted, and graphical sketch of the acquired data.

Site-directed spin labeling EPR is used in structural biology mostly in conjunction with double electron–electron resonance (DEER aka PELDOR) spectroscopy (Figure 1 A) on frozen biomacromolecules.^1^ The freezing procedure, required to obtain the dipolar interaction, hence interspin distances, despite being well tolerated in most cases, poses some limits in terms of analysis of dynamic equilibria in solution, which could be better identified and studied at physiological temperatures. Therefore, in an integrative approach, DEER data on spin-labeled proteins could be complemented using spectroscopic tools that are applicable in the solution state, such as Förster resonance energy transfer (FRET) measurements (two examples can be found here^2,3^). The availability of spectroscopically orthogonal labels increased the information content per sample mostly in conjunction with PDS methods.^4−6^ Moreover, an increased technical sensitivity enabled the detection of frozen spin-labeled biomolecules under physiologically relevant environments^7−11^ and concentrations.^12−16^ To expand the use of PDS methods toward samples at physiological temperatures, immobilized biomolecules carrying sterically hindered labels with long phase memory time can also be employed (for a comprehensive review see ref (17)). Still, frozen or immobilized samples do not allow measuring molecular and solvation dynamics with temporal resolution as can be done at physiological temperatures. Here, we aim to address the possibility to obtain additional information content on orthogonally labeled biomolecules by complementing the structural studies done on frozen samples with solution state investigations. Having at one’s disposal data on the same spin-labeled sample at both cryogenic and physiological temperatures is relevant to corroborate the structural information and unveil complementary properties on biomolecular interactions and equilibria beyond the frozen snapshots.

Here, we will use three room temperature EPR methods on samples carrying nitroxide and gadolinium labels: (i) continuous wave (cw) EPR at X band to monitor nitroxide-labeled side chains dynamics, (ii) ODNP (Overhauser dynamic nuclear polarization) at X band to get information on hydration water around nitroxide probes, and (iii) cw EPR at high field to extract residual Gd–Gd dipolar couplings for distance information (Figure 1B–D).

X-band cw EPR spectroscopy using nitroxide-labeled proteins is a well-established EPR technique to extract dynamics of protein side chains at physiological temperatures (Figure 1B). The specific dynamics of spin-labeled side chains in biomolecules are encoded in their EPR spectral shape, and any change in the rotational correlation time of the spin label will produce a different averaging of the anisotropic hyperfine interactions, leading to distinct patterns of narrowing or broadening of the EPR spectral manifold.^18^ A few studies exist in which gadolinium labels were also employed in the solution state,^19,20^ most recently to “film”-triggered conformational changes of an optogenetic protein.^21^

Overhauser dynamic nuclear polarization (ODNP) is an established method based on the transfer of electron spin polarization from spin label to nearby water ^1^H nuclei via the Overhauser effect^22^ to enhance the ^1^H NMR signal, which has been applied to study water dynamics and changes in water accessibility in the proximity of nitroxide probes (Figure 1C).^23−25^

Finally, cw EPR at high field (240 GHz) at room temperature enables the study of static or time-resolved^21^ residual dipolar interactions between a pair of gadolinium (Gd) labels for distances up to about 4 nm (Figure 1D).^26^

The biomolecular system investigated in this study is a minimal interactome consisting of three proteins from the Bcl-2 family, which are involved in the mitochondrial pathway of apoptosis in human cells. This interactome consists of peptides from a proapoptotic protein (Bim), which inhibit the anti-apoptotic protein Bcl-xL and, depending on their length, can additionally activate the pro-apoptotic protein Bax.^27^ Once activated, Bax oligomerizes at the mitochondrial outer membrane forming a pore through which cytochrome c is released, which marks the point of no return in apoptosis. Site-directed spin labeling (SDSL) EPR techniques, in particular, time-resolved cw EPR at physiological temperatures and distance measurements by pulsed dipolar spectroscopy (PDS) on frozen samples were previously used to study interactions and structural transformation of the Bcl-2 family proteins.^28−30^ The use of orthogonal spin probes has brought additional advances in the PDS analysis of the Bcl-2 interactome by enabling the independent investigation of several DEER channels on the same sample.^31^ Despite the high potential of the previously discussed PDS techniques to solve important questions in structural biology, the strict requirement of freezing or immobilization of biomolecules to extract dipolar information could hinder analysis of nonequilibrium configurations, such as conformational changes triggered by folding or unfolding of proteins, which could be instead identified and possibly followed in time only under physiological conditions.

**Figure 2:**
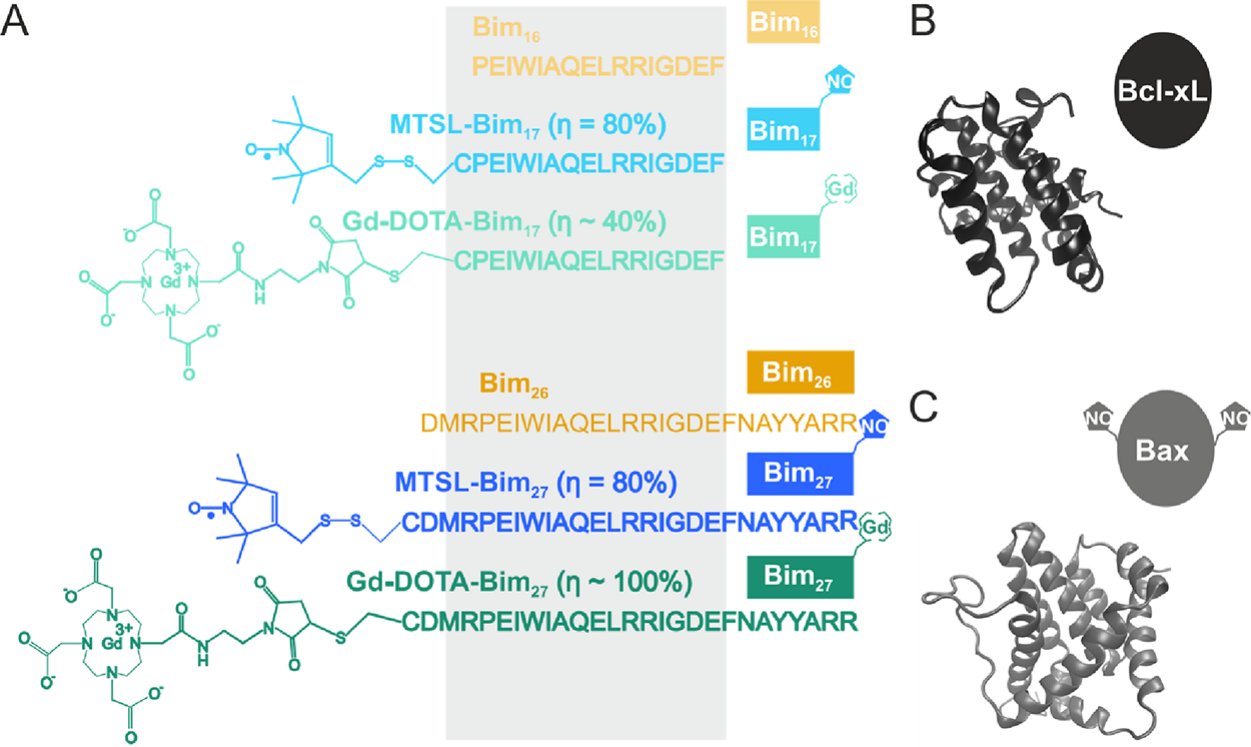
Minimal Bcl-2 interactome investigated. (A) Primary structures of Bim peptides used in this study with the position of the different spin labels highlighted. The spin labeling efficiency (η, spin/peptide concentration) is shown in parenthesis. A schematic color-coded representation of each spin-labeled peptide is shown on the right. The size of the peptides is encoded in the length of the colored boxes representing them. In the following, the Gd-Bim peptides will be named “Gd-peptides” for simplicity. (B) Ribbon representation of C-terminally truncated Bcl-xL (PDB: 4QVF) and (C) full length Bax (PDB: 1F16). Insets show a schematic representation of the unlabeled Bcl-xL used in this study and of the spin-labeled Bax carrying the nitroxide labels at the two natural cysteines 62 and 126.

Here, we use Bim peptides spin-labeled with nitroxide (MTSL) or gadolinium (Gd-DOTA maleimide) spin labels (Figure 2A)^29^ in combination with unlabeled or MTSL-labeled Bcl-2 protein partners (Bax or Bcl-xL) (Figure 2B,C). We provide insights into the peptide-induced activation of Bax, the peptide-induced inhibition of Bcl-xL, and the self-interaction and water accessibility of peptides in the presence and absence of Bcl-xL at physiological temperature using EPR methods. The data acquired here at physiological temperatures complement and corroborate the information previously obtained on the same Bim-Bcl-xL system at cryo-temperatures,^29^ thereby showcasing approaches to increase the information content for future structural biology studies.

## Results and Discussion

Bim peptides of different lengths have different properties, in terms of self-interaction, Bax activation, or interaction with Bcl-xL, as described previously.^27^ The long Bim peptides used in this study (Bim_26_) form homo-tetramers in solution (PDB: 6X8O), and we confirmed that the spin-labeled variants (Gd-Bim_27_ and MTSL-Bim_27_) retain the possibility to self-interact in frozen solutions.^29^ The long Bim_26–27_ peptides (spin-labeled or not) activate Bax and inhibit Bcl-xL. Intriguingly, the short Bim_16–17_ peptides (spin-labeled or not) do not self-interact and have only minor effects on Bax activation but retain the ability to bind to Bcl-xL, inhibiting its anti-apoptotic function.^29^

When nitroxide-labeled Bax is activated by proapoptotic proteins or peptides (e.g. Bim, Bid), it transforms from a water-soluble monomer into a membrane-embedded oligomer. This transformation was monitored by time-resolved X-band EPR via a decrease in the spin label mobility of the two nitroxide-labeled natural cysteines of Bax (positions 62 and 126).^32^ Here, we show that we can monitor the dynamic transformation of nitroxide-labeled Bax activated by Gd-labeled Bim peptides at physiological temperature, without spectral disturbances on the nitroxide response caused by the orthogonal Gd spins present in the sample.

**Figure 3:**
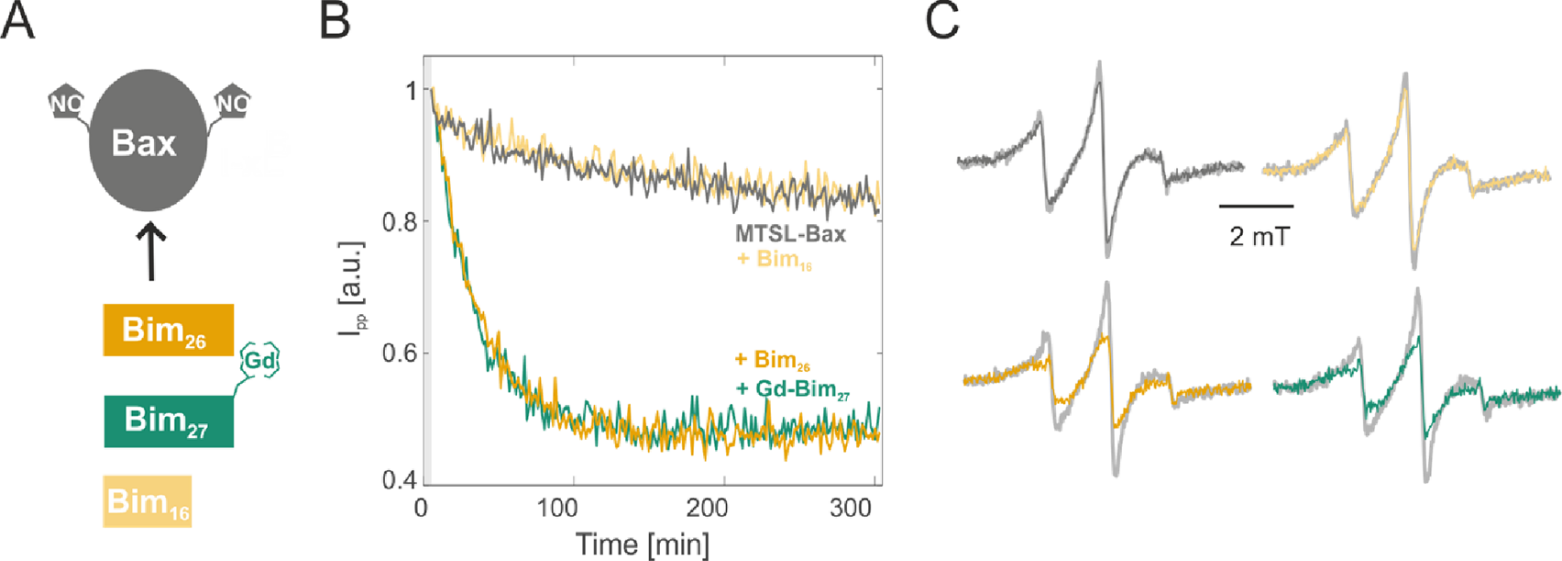
Nitroxide kinetics in the presence and absence of Gd-peptides at physiological temperature. (A) Schematic representation of the interaction partners. (B) Cw EPR kinetics at 37 °C of nitroxide-labeled Bax in LUVs with a composition mimicking the outer mitochondrial membrane alone and with different Bim peptides. Bax was added at 20 μM final concentration, the peptides were added in a 1:1 stoichiometric ratio. (C) Cw EPR spectra extracted from the kinetic trace of panel B after 5 min (light gray) and 305 min (colored) incubation.

Nitroxide-labeled Bax in the presence of large unilamellar vesicles (LUVs) was incubated with Bim_16_, Bim_26_, or Gd-Bim_27_, and the peak-to-peak intensity of the central EPR line was plotted versus time (Figure 3). When monitoring the cw EPR signal of nitroxide-labeled Bax that gets activated by Bim or another proapoptotic protein (e.g. Bid^28,32^), we expect a decrease in the intensity of the central line, induced by a decreased rotational motion going from the inactive Bax monomer to the active membrane-embedded oligomer.^32^ Bax in the presence of LUVs already exhibits a minor intensity decay over time (dark gray in Figure 3B) due to its known partial auto-activity to form oligomers.^29^ The presence of the Bim_16_ in the mixture (yellow in Figure 3B) does not modify the autoactivity of Bax, as expected from the inability of the short peptides to activate Bax. However, when Bim_26_ or Gd-Bim_27_ are present, a prominent exponential signal decay is observed (orange and green in Figure 3B and Figure S1), indicating that the long peptides (spin-labeled or not) promote Bax insertion into LUVs. The unperturbed kinetics of nitroxide-labeled Bax obtained in the presence of Gd-Bim demonstrates that it is possible to measure kinetics by cw EPR of the nitroxide label in the presence of orthogonal Gd-DOTA label in the sample. This is feasible because the X-band cw EPR spectrum of Gd-DOTA at physiological temperatures is below the detection limit due to the large width of their spectra. In fact, the commercially available Gd-DOTA labels used here have a ∼700 MHz zero-field splitting (ZFS)^33^ and are therefore characterized by ∼10 mT linewidths at X band. Therefore, we can conclude that the Gd-DOTA-labeled peptides at X band are EPR “silent” if concentrations are kept below 100 μM and the kinetics of the nitroxide-labeled protein induced by a Gd-DOTA-labeled peptide can be extracted without loss of sensitivity. Notably, other Gd-chelators with smaller ZFS might become detectable at such concentrations (see for example^19^) and their possible effects should be taken into consideration.

**Figure 4:**
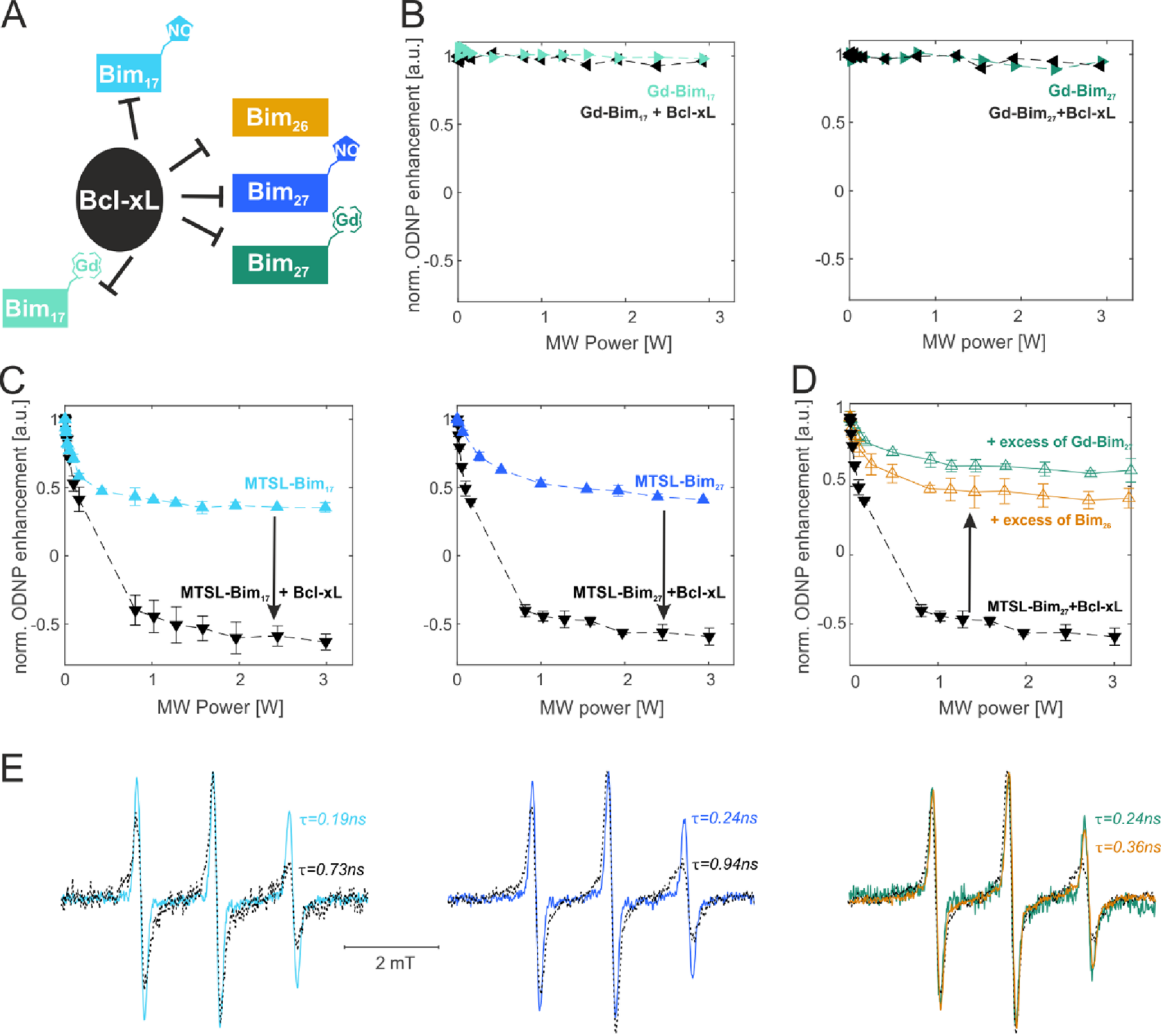
Room temperature ODNP on orthogonally labeled samples. (A) Schematic view of the probed interactions. (B) ODNP enhancement obtained at room temperature in aqueous solution in the presence of Gd-Bim peptides alone (final concentration 15 μM) or in the presence of Bcl-xL (1:1 stoichiometry). (C) ODNP enhancement obtained at room temperature in aqueous solution in the presence of MTSL-Bim peptides alone (final concentration 15 μM) or in the presence of Bcl-xL (1:1 stoichiometry). (D) Competition experiments performed with the MTSL-labeled peptides and Bcl-xL (1:1 stoichiometric ratio) upon addition of excess unlabeled or Gd-labeled Bim peptides in a 1:3 ratio with respect to the MTSL-peptide. Cw X-band EPR spectra detected on the ODNP samples presented above, respectively. Rotational correlation times were extracted using the approximation described in ref (35).

The antiapoptotic Bcl-xL protein inhibits Bax activation by sequestering Bim peptides. All spin-labeled Bim peptides in Figure 2A were shown to retain the ability to bind Bcl-xL in frozen aqueous solution using DEER.^29^ Here, we extract information on Bim-Bcl-xL interactions (Figure 4A) at room temperature using Overhauser dynamic nuclear polarization (ODNP) (Figure 4 and Figure S2).

There are no ODNP effects induced by excitation of the Gd EPR transitions on the ^1^H NMR signal; therefore, the Gd-Bim peptides are ODNP silent (Figure 4B). In contrast, the free peptides MTSL-Bim_17_ and MTSL-Bim_27_ at 15 μM (spin) concentration showed an ODNP-modulated signal amplitude of +0.5 relative to the thermally polarized ^1^H NMR signal (+1.0) at 3 W microwave power (Figure 4C). The ODNP-modulated ^1^H NMR signal amplitude by both MTSL-Bim_17_ and MTSL-Bim_27_ reduced to −0.5 (i.e., ^1^H NMR signal inversion and reduction in signal amplitude) in the presence of the unlabeled Bcl-xL. This distinct change in the water signal enhancement indicates binding of both peptides to the antiapoptotic protein, Bcl-xL. Both MTSL-Bim_17_ and MTSL-Bim_27_ are small peptides that tumble freely in solution and whose MTSL label experiences rapid dynamics and short relaxation times. Hence, MTSL-Bim_17_ and MTSL-Bim_27_ exhibit a sharp three-line cw EPR spectrum with lineshape displaying rotational correlation times of ∼0.19 and 0.24 ns, respectively (Figure 4E). The signal inversion seen with ODNP upon incubation with Bcl-xL signifies greater ODNP effects that can originate from faster water dynamics, greater water accessibility, and/or greater saturation of the MTSL nitroxide label. However, given that Bim is a short peptide with MTSL fully water exposed, it is unlikely that the water accessibility and/or water dynamics at the Bim peptide surface would increase upon binding to Bcl-xL. Binding of Bim to Bcl-xL is directly confirmed by the change in its cw X-band spectral line shape that are consistent with the emergence of slower rotational correlation times of ∼0.73 and 0.94 ns, respectively (Figure 4E). Therefore, the observed enhancement in the ODNP effect that resulted in the inversion of the ^1^H NMR signal of water near MTSL attached to bound Bim peptides compared to free Bim peptides is due to a change in the saturation factor of the EPR transitions. The motion-dependent saturation factor is well known in the literature,^23,34^ and the saturation factor changes from ∼1/3 for fast tumbling peptides in solution to ∼1 for peptides bound to their target protein. Such a change in saturation increases the ODNP enhancement upon protein binding and can therefore be efficiently used as a fingerprint of the binding of spin-labeled peptides or other fast tumbling small drugs/ligands to a protein partner. In addition, the enhancement obtained with the nitroxide labels located in the short and long peptides bound to Bcl-xL provides indication on the water accessibility of the nitroxide side chain. We found very similar ODNP enhancements (∼−0.5) for both protein-bound peptides, indicating a similar water accessibility of the spin-labeled N-terminal region of the peptides in complex with Bcl-xL.

Using ODNP, we could additionally prove that excess of Gd-Bim_27_ peptides (ODNP silent) can compete out the MTSL-Bim_27_ peptides from their binding pocket in Bcl-xL as efficiently as the unlabeled Bim_26_ (Figure 4D). In fact, in the presence of excess competitors, the ODNP traces of MTSL-Bim_27_ in the presence of Bcl-xL went back to the values of free MTSL-Bim_27_, indicating an efficient release of the MTSL-peptides from Bcl-xL into the bulk solution. The results are corroborated by the cw EPR spectra (Figure 4E). ODNP can therefore be performed on nitroxide-labeled peptides in the presence of orthogonally labeled partners because the Gd-labeled peptides are ODNP silent. The competition experiments showcase that neither the Gd- nor the MTSL-labels alter the function of Bim peptides in the Bcl-2 protein interactome.

As discussed above, Gd-DOTA complexes are silent in X-band cw EPR at room temperature and micromolar concentrations and do not interfere with the nitroxide spectral shape. The spectroscopic properties of the two spins change at high magnetic fields, as the linewidth of the Gd −1/2 to 1/2 central transition gets narrower, while the spectral width of nitroxides increases tremendously due to *g* anisotropy. Based on the *g*, *A*, and ZFS parameters of MTSL and Gd-DOTA,^1^ there is only a negligible spectral overlap between the two orthogonal labels at 240 GHz at physiological temperature even considering rotational correlation times approaching the rigid limit (Figure S3). Therefore, MTSL and Gd-DOTA can be considered spectroscopically orthogonal at room temperature at high frequency/field (240 GHz/8.6 T) and Gd-labeled proteins can be selectively detected with high field cw EPR without disturbance arising from the nitroxide spectra. Further, the Gd^3+^ magnetic moment is sufficiently large (7 times larger than spin 1/2), and the Gd-DOTA central transition is sufficiently narrow, in which dipolar coupling between pairs of nearby Gd-DOTA labels separated by <4 nm can lead to observable line broadening at room temperature.^26^

**Figure 5:**
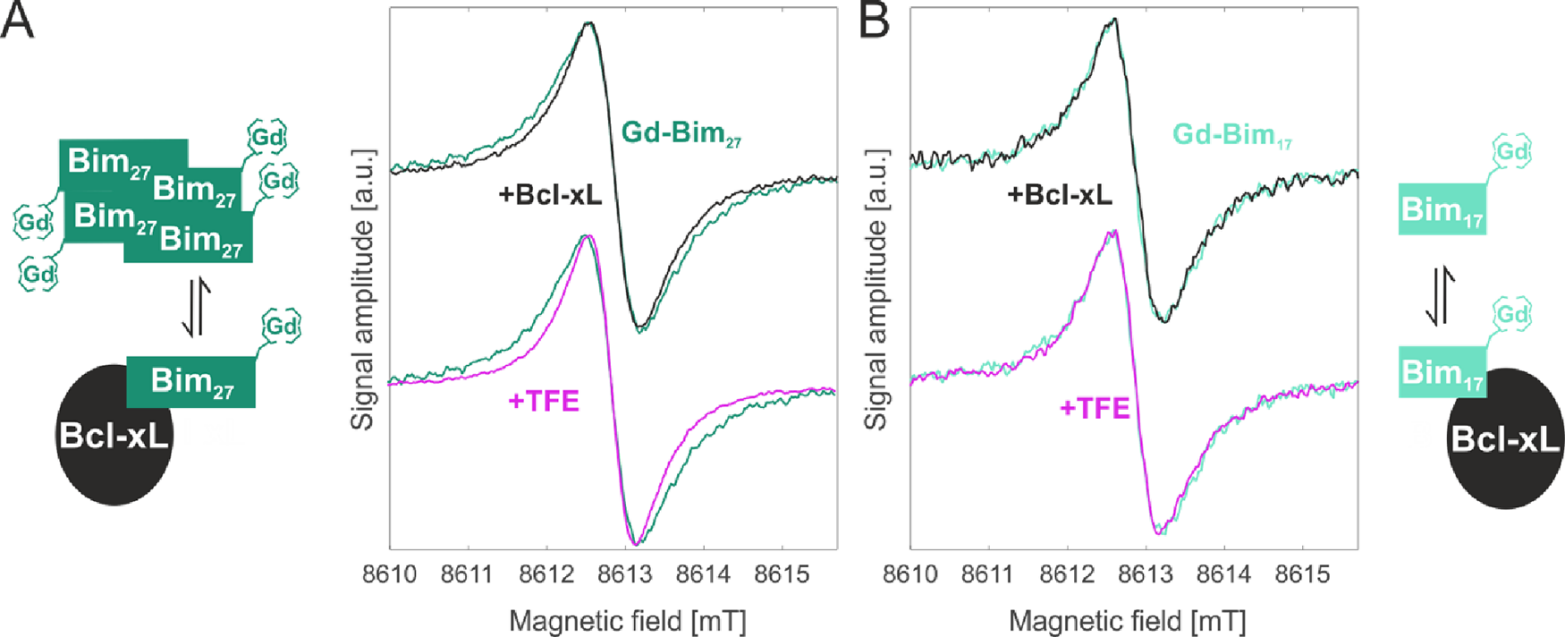
“Room temperature” (288 K) Gd–Gd distances by high field cw EPR. (A) Schematic representation of the interactions and cw EPR spectra of the central −1/2 to +1/2 transition detected on the Gd-Bim_27_ peptides in solution (final concentration 100 μM) in the absence or in the presence of Bcl-xL (1:1 stoichiometric ratio) or in the absence and presence of TFE (66% v/v). (B) Schematic of the interactions and cw EPR spectra on the Gd-Bim_17_ peptides under the same experimental conditions. The spectra detected at cryogenic temperatures are shown in Figure S4.

To demonstrate this, we selectively monitored the interaction between Gd-Bim_27_ with Bcl-xL at 8.6 T (Figure 5), extracting the residual dipolar interactions present in the gadolinium spectra at room temperature due to changes in the oligomerization of Gd-Bim_27_.^26^ Low temperature DEER measurements already showed that Gd-Bim_27_ peptides in aqueous solution form homo-tetramers characterized by a broad distance distribution ranging from 2 to 6 nm.^29^ These oligomers can be destroyed by the addition of Bcl-xL, as shown by DEER, proving that the heteromeric Bim-Bcl-xL interaction is stronger than the Bim self-interactions. In the spirit of this paper, we next test whether these interpeptide interactions can also be probed at physiological temperature by cw high-field EPR of Gd^3+^. The high field Gd spectra detected at room temperature in a solution of Gd-Bim_27_ peptides were found to be broader in the absence than in the presence of Bcl-xL (green vs black in Figure 5A). This effect can be explained by the dipolar broadening present in the tetrameric Gd-Bim_27_ peptide oligomers, which is eliminated by the addition of Bcl-xL that breaks the tetramers apart. To prove that the line broadening is due to the disruption of the tetramers, we added TFE (2,2,2-Trifluorethanol) to the solution (magenta in Figure 5 A), which was previously shown to break apart the Bim tetramers.^29^ Indeed, we detected the expected decrease in linewidth in the presence of TFE (green vs magenta in Figure 5A).

To further validate the dipolar origin of the observed linewidth effects of the long Gd-Bim_27_ peptides, we used as a control the short Gd-Bim_17_ peptides under the same experimental conditions. Since the short peptides are monomers in all conditions, no changes upon addition of Bcl-xL or TFE were expected. In agreement with the predictions, no spectral changes were observed (Figure 5B), corroborating the notion that the residual dipolar broadening due to inter-Gd interactions within Gd-Bim_27_ tetramers can be detected at room temperature and the Bcl-xL-induced dissociation of the tetramers can be monitored in the solution state.

## Conclusions

In conclusion, this is a case study in which different room temperature EPR techniques are applied to study interactions of ligands to their target proteins in solution state using orthogonal labels. We demonstrate that cw X-band EPR (static or time-resolved) of nitroxide-labeled molecules can be performed with no perturbation induced by the Gd-labeled molecules in the sample. In addition, we demonstrated the applicability of ODNP to study the binding of small ligands to their target proteins at micromolar concentrations on very small sample volumes (a few microliters) via changes in the saturation factor that serve as a fingerprint of ODNP efficiency and spin label’s molecular dynamics. The Gd spins are silent in ODNP experiments; therefore, Gd-labeled proteins can be safely used in the presence of a nitroxide-labeled protein partner to study interprotein interactions. The Gd spins, which are “silent” both in cw X-band EPR and ODNP at physiological temperatures, yield narrow spin −1/2 to 1/2 transitions at very high magnetic fields and hence are sensitive to Gd–Gd distances <4 nm in solution. Due to orthogonal spectral separation between Gd and nitroxide, the Gd–Gd distances can potentially be measured also in the presence of nitroxide-labeled partners in the same sample. The EPR methods discussed here provide information about molecular dynamics, water accessibility, and interspin label distances at physiological temperatures. In the future, these measurements of dynamics can be easily combined with pulsed dipolar spectroscopy methods performed on samples at cryogenic temperatures to increase the information content per sample and to strengthen the physiological relevance of the EPR-based findings.

## Supporting Information Available

Materials and methods and additional experimental details, including figures of primary data and simulated high field nitroxide-gadolinium spectra (PDF)
