# Supplementary material for: Spectroscopically Orthogonal Spin Labels in Structural Biology at Physiological Temperatures
Source: J Phys Chem B. 2023 Jul 25;127(30):6668–74. doi: 10.1021/acs.jpcb.3c04497 (PMC10405217; doi:10.1021/acs.jpcb.3c04497)
Supplement: Supplementary file 1 — jp3c04497_si_001.pdf [file jp3c04497_si_001.pdf]

Spectroscopically Orthogonal Spin Labels in Structural Biology at Physiological Temperatures

MarkusTeucher,[†] SvetlanaKucher,[†‡] M. HadiTimachi,[†] C. BlakeWilson,[§∥] DariuszŚmiłowicz,[†] RaphaelStoll,[†] NilsMetzler-Nolte,[†] Mark S.Sherwin,[§∥] SongiHan,[∥⊥] EnricaBordignon[*†‡]

†Faculty of Chemistry and Biochemistry, Ruhr University of Bochum, Bochum 44801, Germany

‡Department of Physical Chemistry, University of Geneva, Genève 1211, Switzerland

§Department of Physics, University of California, Santa Barbara, Santa Barbara, California 93106, United States

∥Institute for Terahertz Science and Technology, University of California, Santa Barbara, Santa Barbara, California 93106, United States

⊥Department of Chemistry and Biochemistry, University of California, Santa Barbara, Santa Barbara, California 93106, United States

Supplementary information

MATERIALS AND METHODS

Protein preparation

All details related to the solid state peptide synthesis and the spin labeling procedures are published as supplementary material in[1]. The peptide concentration was determined by UV/vis spectroscopy (Eppendorf BioSpectrometer Basic) using a 280 nm extinction coefficient of 8480 M^{-1} cm^{-1} for the long Bim (26-27aa) peptides and 5500 M^{-1} cm^{-1} for the short Bim peptides (16-17aa). Full length human Bax was expressed in *E. coli*, purified, and labelled with MTSL at the two natural cysteines C62 and C126 as described in[2,3]. The concentration was determined by UV/vis using an extinction coefficient of 36940 M^{-1} cm^{-1} [4].The Bcl-xL protein used in this study lacks the C-terminal helix 9 and was expressed and purified as described previously[1]. The concentration was determined by UV/vis using a theoretical extinction coefficient of 47440 M^{-1} cm^{-1} calculated via ExPASy program (https://www.expasy.org/).

CW EPR kinetics measurements

All samples were prepared at a final volume of 20 µL, containing 4 µL of large unilamellar vesicles (LUVs) and a variable protein/peptide volume to reach a final concentration of 20 µM for each reaction partner. The void volume to reach 20 µL was filled using SMART buffer (20 mM, NaCl 150 mM, pH 7.5). Subsequently, the sample was placed in a glass capillary (Blaubrand) and sealed with Critoseal (VWR). CW EPR kinetics were recorded using an Elexsys E580 X-band (9.45GHz) spectrometer (Bruker, Germany) equipped with an ER 4122 SHQ (Bruker, Germany) and a nitrogen flow cryostat. The temperature was stabilized with a temperature controller (Eurotherm) at 37°C. The following measurement parameters were used: mod. amplitude – 0.15 mT, mod. frequency – 100 kHz, sweep width – 10 mT, sweep time – 20.5 s, microwave power 9.5 mW.

ODNP

ODNP measurements were performed on the home-made ODNP setup at RUB. The setup consisted of an Elexsys E580 X-band spectrometer equipped with a SHQ cavity (Bruker), a microwave power source Bridge12 (Bridge12), a NMR Avance III HD spectrometer (Bruker) and a home-made NMR coil based on S. Han design. ODNP measurements were performed using an automated home-developed Python package "TopDNP" running from Top- Spin software (Bruker). The code with description of usage is freely available on GitHub: https://github.com/haditim/TopDNP. ODNP data were analyzed using an automated home-developed Python package "DNPy", the code with the detailed information about the evaluation procedure is free available on https://github.com/haditim/DNPy.

For ODNP measurements 4µL of corresponding BIM-peptide (C_{final}=15µM (spin)) or its mixture with BclXL (1:1 protein:protein) was transferred into pre-cut 0.6 mm ID x 0.84 mm OD round quartz capillary (VitroCom) and sealed with Critoseal (VWR) from both sides. For competition experiment, BIM-peptide pre-mixed and pre-incubated for 1h at room temperature was mixed with BIM_{26} or Gd-BIM_{27} in 1:3 ratio. The same samples in capillaries were put into a bigger 3 mm OD quarz capillary (Quarzglas Heinrich) for the subsequent CW EPR measurements on a Miniscope MS5000 (Magnettech, Freiberg Instruments) using 10 mW microwave power and 0.1 mT modulation amplitude (100 kHz mod. frequency) at room temperature.

240 GHz CW EPR

All room temperature samples were prepared at a final volume of 6 µL with a variable protein/peptide volume to reach a final concentration of 100 µM for each reaction partner. The void volume to reach 6 µL was filled depending on the sample type with SMART buffer and/or TFE (2,2,2-Trifluorethanol). The low temperature samples (30 K) were prepared by at a higher sample volume of 9 µL due to the added 33% final glycerol-d_8 concentration. Accordingly, these samples have a 67 µM final protein/peptide concentration.

The high-field cw EPR experiments were carried out using a home-built 240 GHz EPR spectrometer at the Institute for Terahertz Science and Technology at UCSB described previously[5,6]. The spectrometer uses a 55 mW, 240 GHz solid-state microwave source (Virginia Diodes Inc.) together with a 12.6 T field-sweep capable superconducting magnet (Oxford Instruments) with a room temperature bore, housing a home-built EPR probe contained inside a continuous flow cryostat (Janis Research Company). Field-swept cw EPR experiments were

carried out at a magnetic field near 8.61 T. Microwaves were quasi-optically coupled into a 1.25 mm corrugated waveguide to the sample position, and the reflected EPR signal was received in induction mode. Lock-in detection with field modulation supplied by a modulation coil was used, with a modulation amplitude of 0.15 mT and a 20 kHz modulation frequency. A sweep width of 50 mT was typically used, with a 0.1 mT/s sweep rate. All samples were inserted in rectangular flat 100 μm thick, 2 mm by 5 mm borosilicate glass capillaries (VitroCom) by taking advantage of the capillary forces and sealed on both ends with wax. The effective volume inside of the flat quartz capillary was in the order of 1 μL. Glass capillaries were placed on a Teflon tape-covered silver mirror. No resonator was used[7]. "Room temperature" measurements were carried out at 288 K, the ambient temperature inside the continuous flow cryostat. Low-temperature measurements were carried out at 30 K using cold helium gas. The sample temperature was monitored using Cernox temperature sensor (Lakeshore Cryogenics Inc.) mounted at the end of the corrugated waveguide near the sample position.

Supplementary Figures

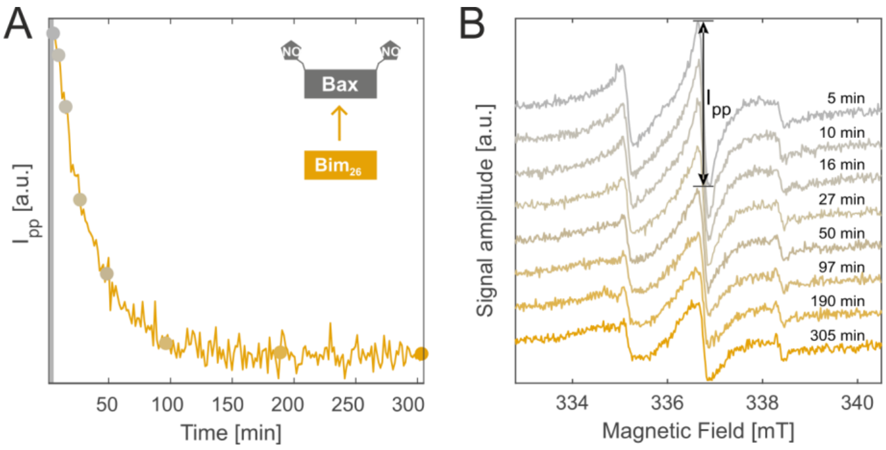

Figure S1. **Cw EPR kinetics**. (A) Plot of the peak-to-peak intensity vs. time of the central line of the cw EPR spectra of spin-labeled Bax in LUVs in the presence of Bim_{26}. (B) Selected cw EPR spectra at different time points. The arrow shows the peak-to-peak intensity plotted in (A).

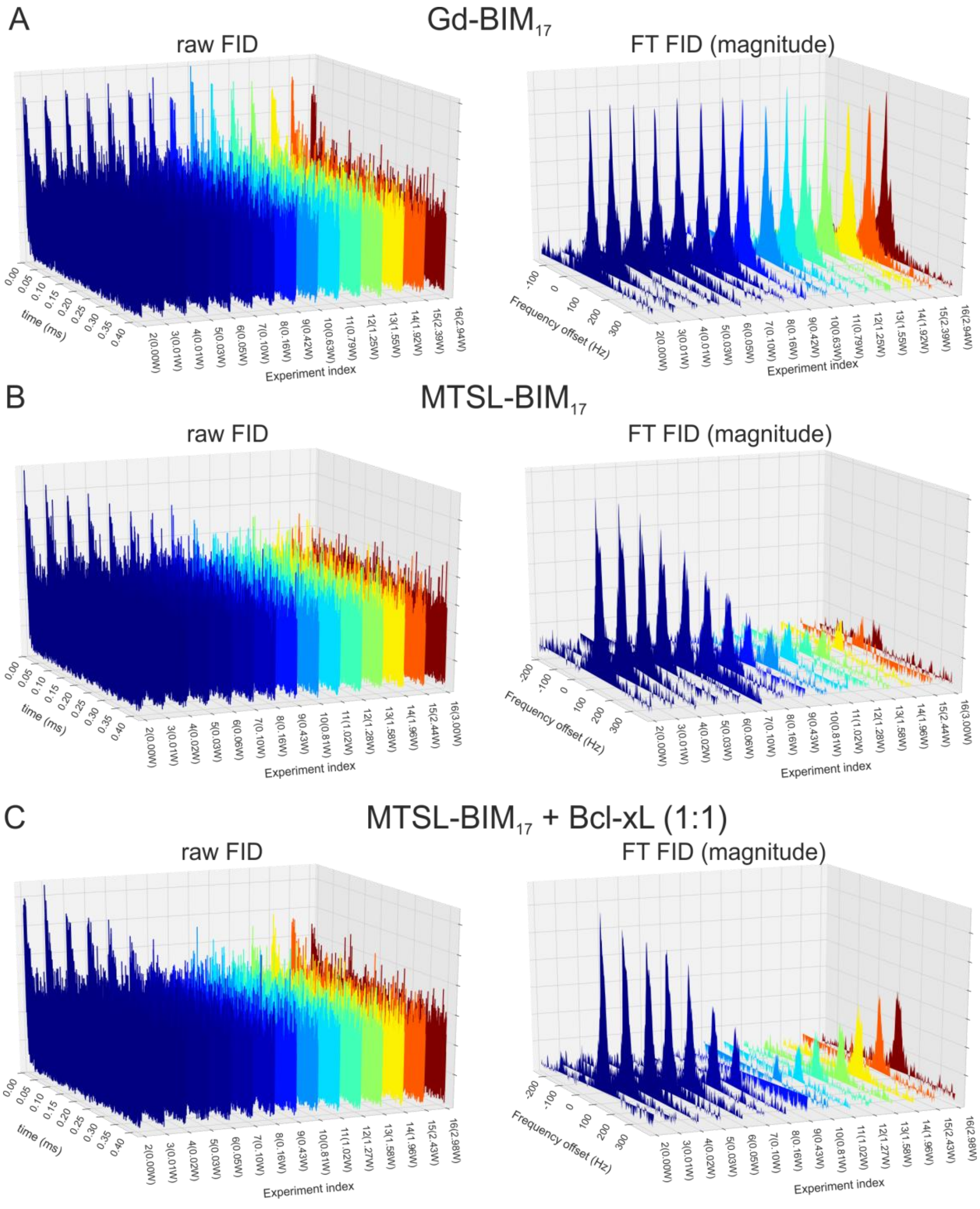

Figure S2. **ODNP primary data**. Representative data for Gd-BIM$_{17}$(A), MTSL-BIM$_{17}$ (B) and MTSL-BIM$_{17}$ – Bcl-xL (C) related to Fig. 4C in the main text. Left panel: primary FID traces after digital filter removal and offset correction. Right panel: FT FID (magnitude) of the traces in the left panel used for the integral enhancement calculation.

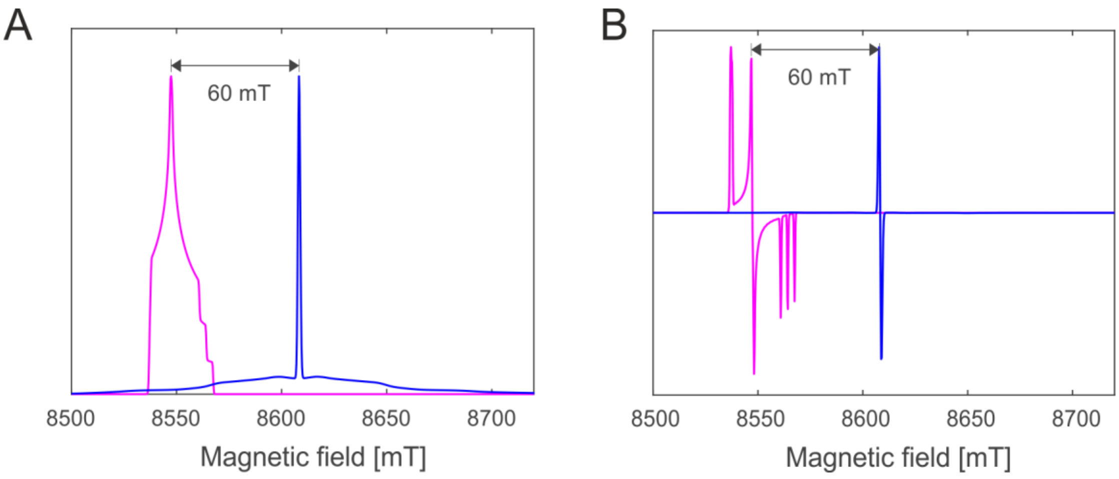

Figure S3. **High field spectral simulation for nitroxide and gadolinium labels.** Powder spectra of Gd-DOTA and MTSL simulated with the EasySpin[8] pepper subroutine at 240 GHz showing the spectral selectivity at high field in the frozen state, which also exemplificative of the limiting case in which the rotational correlation times approach the rigid limit at room temperature. A: Absorption spectra (harmonic 0) and B: derivative spectra (harmonic 1). The following parameters were used (as in[9]): MTSL g = [2.0085, 2.0061, 2.0022], A = [13, 13, 92] in MHz, lw = 0.5 mT; Gd-DOTA[10] g_{iso} = 1.992, D = [715 150] in MHz, D_{strain} = 200 MHz, lw = 1 mT.

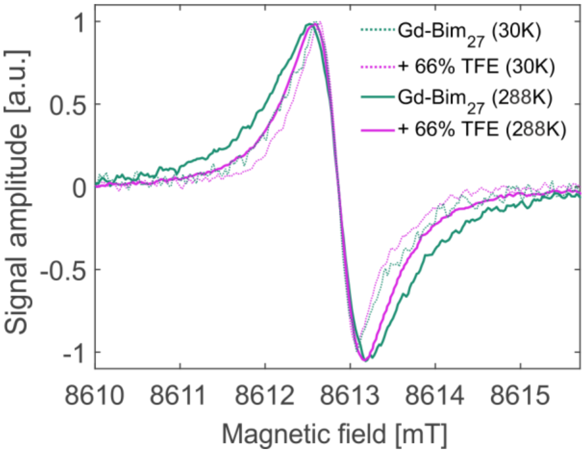

Figure S4. **High-field cw EPR spectra at cryogenic temperature.** Comparison of the 240 GHz cw EPR spectra acquired at room temperature (from Fig. 5) and at 30 K. The spectra are narrower at low temperature, but the addition of TFE is shown to decrease the linewidth both at room and at 30 K due to the disappearance of the dipolar broadening arising from inter-peptide interactions.

REFERENCES

1 Assafa, T. E. *et al.* Biophysical Characterization of Pro-apoptotic BimBH3 Peptides Reveals an Unexpected Capacity for Self-Association. *Structure* **29**, 114-124 (2021).

2 Bleicken, S. *et al.* Molecular Details of Bax Activation, Oligomerization, and Membrane. *Journal of Biological Chemistry* **285**, 6636-6647 (2010).

3 Bleicken, S. *et al.* Structural Model of Active Bax at the Membrane. *Molecular Cell* **56**, 496-505 (2014).

4 Teucher, M. *et al.* A new perspective on membrane-embedded Bax oligomers using DEER and bioresistant orthogonal spin labels. *Scientific Reports* **9**, 13013 (2019).

5 Edwards, D. T. *et al.* Extending the distance range accessed with continuous wave EPR with Gd3+ spin probes at high magnetic fields. *Phys Chem Chem Phys* **15**, 11313-11326 (2013).

6 Takahashi, S. *et al.* Pulsed electron paramagnetic resonance spectroscopy powered by a free-electron laser. *Nature* **489**, 409-413 (2012).

7 Maity, S. *et al.* Triggered Functional Dynamics of AsLOV2 by Time-Resolved Electron Paramagnetic Resonance at High Magnetic Fields. *Angew Chem Int Ed Engl*, e202212832 (2023).

8 Stoll, S. & Schweiger, A. EasySpin, a comprehensive software package for spectral simulation and analysis in EPR. *J Magn Reson* **178**, 42-55 (2006).

9 Galazzo, L., Teucher, M. & Bordignon, E. Orthogonal spin labeling and pulsed dipolar spectroscopy for protein studies. *Methods Enzymol* **666**, 79-119 (2022).

10 Clayton, J. A. *et al.* Quantitative analysis of zero-field splitting parameter distributions in Gd(iii) complexes. *Phys Chem Chem Phys* **20**, 10470-10492 (2018).
